# Analysis of Active and Passive Deformation of Expanded Polystyrene Foam under Short-Term Compression

**DOI:** 10.3390/ma15217548

**Published:** 2022-10-27

**Authors:** Saulius Vaitkus, Sigitas Vėjelis, Jurga Šeputytė-Jucikė, Sylwia Członka, Krzystof Strzelec, Agnė Kairytė

**Affiliations:** 1 Laboratory of Thermal Insulating Materials and Acoustics, Institute of Building Materials, Faculty of Civil Engineering, Vilnius Gediminas Technical University, Linkmenu St. 28, LT-08217 Vilnius, Lithuania; 2 Institute of Polymer & Dye Technology, Lodz University of Technology, 90-924 Lodz, Poland

**Keywords:** active and passive deformation, expanded polystyrene, plastic deformation, short-term compression

## Abstract

In this paper, we undertake a detailed analysis of the active and passive deformation of expanded polystyrene (EPS), which is used as a thermal insulating layer in building partitions, under short-term compressive loading. The values of residual strain in 10–40 kg/m^3^ density EPS after monotonically increasing loading under active deformations of 20%, 30%, 40%, 50%, and 60% with the following complete removal are determined. These values are a physical sign of the elastic–plastic state of EPS. It has been shown that the final destruction of cells takes place in EPS when the active strain reaches 50%. Empirical equations are proposed to estimate the residual strain of EPS based on density with determination coefficients varying from 0.744 to 0.986 at a confidence level of 90%. Moreover, graphical interpretations with regression equations for residual strain dependence on density and compressive strength, as well as density and active strain, were proposed with determination coefficients equal to 0.779 and 0.717, respectively.

## 1. Introduction

Expanded polystyrene (EPS) slabs are widely used in the building industry as thermal insulating layers [1,2]. Their main advantages are lightness, good heat and sound insulation properties, and relatively high relative strength. The mechanical performance of foamed materials has been widely investigated in the literature [3,4,5]. Gibson and Ashby [6] provide a thorough discussion of the mechanical performance of various cellular solids, and many scientific studies have reported that the mechanical properties of foams are intrinsically determined by the matrix and the cell microstructures [7,8,9]. The strength and deformation properties of EPS slabs, together with their thermal insulation properties, are the most important characteristics in determining their use in the building construction practice. Moreover, the properties of polymeric foams can be easily improved by controlling the pore size, apparent density, and cell structure and with the addition of various modifiers [10,11]. Additionally, Bai et al. [12] observed that the cell size of the polystyrene foam and its apparent density could be optimized by controlling the foaming conditions. Kairytė et al. [13] showed that the addition of titanate-grafted paper production waste particles into polyurethane foam could improve not only the mechanical performance but also the water resistance and thermal insulating properties. Additionally, Marsavina and Sadowski [14] showed that the impregnation of such polymeric foam materials by 26% improves the dynamic fracture property.

Further on the mechanical response, Vilau and Dudescu [15] presented the results of three-point compression and bending tests for four types of EPS with varying densities at different speeds and proposed a theoretical mathematical model that can be implemented to determine the theoretical mechanical characteristics as well as to model the EPS foam properties in numerical simulations. Meanwhile, Liu et al. [16] presented a model involving five elements that are considered as a function of the apparent density for compressive loading. Additionally, Ozturk and Anlas [17,18] showed the changes in deformation under compressive loading and unloading and suggested a model and a method to predict the force, absorbed energy, and deformation. The latter study was followed with a further investigation [19], which concluded a finite element simulation of multiple compressive loading and unloading cycles of EPS foam for packaging purposes. Even though the suggested models are quite accurate, Stoia et al. [20] presented the experimental data obtained for the mixed-mode fracture properties of polyamide and concluded that underlying differences between theoretical models and experimental results exist. According to Khristenko et al. [21], the main reason is that the produced materials and/or products usually contain random imperfections that occur during production, e.g., molding, casting, 3D printing, etc. These imperfections have a significant impact on the material properties and can result in some uncertainties during the mechanical response.

EPS under simple compression has elastic properties, and residual deformation only occurs at a certain stage of loading [6]. An overwhelming majority of intended uses and corresponding scientific investigations of EPS focus on its elastic–plastic properties, including the relaxation and cyclic loading processes [22,23,24]. EPS deformation mechanisms under various high loadings have also been studied [25,26].

An important feature of the deformation beyond the elastic limit is the nature of the discharge, such as the process in which the external forces change and in which all areas of the material that experience deformation also experience decreased stress. Unfortunately, only few studies [26,27] have focused on the residual deformation of EPS.

However, deformation properties under transient compressive loads (residual deformations) of non-modified EPS have not yet been sufficiently investigated [28]. The results of these properties are essential for a rational decision on the optimal use of the products. The extensive applications of EPS have stimulated research on their deformation process, for example, the stress and strain at any point of loading have been studied to determine the residual strain after a full or partial removal of the load.

The aim of the present investigation is to study the deformation process of EPS under short-term compression. In order to achieve the aim, the determination of compressive stress under 15–65% strain of 10–40 kg/m^3^ EPS slabs was conducted, and respective regression models to determine residual deformation were suggested. Additionally, scanning electron microscopy (SEM) images of the deformed samples were presented for graphical visualization of EPS under compressive loading at different deformations.

## 2. Materials and Methods

### 2.1. Materials

An experimental study of the deformation of EPS under short-term compression was performed on slabs made by foaming a raw material in the form of beads—rigid granules 0.8–2.5 mm in diameter, which are produced by the leading European companies Styrochem and BASF. All the EPS slabs were produced according to EN 13163 [29] requirements in different expanded polystyrene plants in Lithuania.

### 2.2. Methods

Tests were carried out on cubic specimens with an edge length of 50 ± 1 mm, which were cut from slabs with a nominal thickness of 50 mm that are the most typical thermal insulation products for building purposes. The dimensions of the specimens were measured according to EN 12085 [30], and the density of the specimens was determined according to EN 1602 [31]. The initial characteristics of the specimens are presented in Table 1.

Short-term compression of the specimens was performed on a computerized testing machine H10KS (Hounsfield, UK); the compression conducted was unconfined. The experimental samples (see Table 1) were deformed to set strain values ranging from 15 to 65% (by steps of 5% estimated with an accuracy not exceeding ± 0.5%), and the stress required to maintain these strain values was measured. The maximum load–cell capacity of the testing machine was 10 kN. The loading rate was $0.1\times d_{s}$ mm/min, where $d_{s}$ is the specimen thickness in mm according to EN 826 [32]. Until the stress and strain are monotonically increasing, the relationship between $\sigma_{i}$ and $\varepsilon_{i}$ was registered by a diagram of elastic–plastic deformation. Upon reaching the fixed strain $\varepsilon_{i}$, the load was removed completely. The absolute change in the measuring base of the specimen was measured with a linear gauge (Ozaki D-100S) with a resolution of 0.001 mm and an accuracy of 0.005 mm. Registration was performed immediately after the complete removal of the load, then after 24, 72, 144, 288, and 600 h, and thereafter, at 30, 60, and 90 days and at the end of the experiment. The residual (plastic) deformation $\varepsilon_{pl}(t_{i})$ after the complete removal of the compressive stress at the time of “rest” $t_{i}$ for each specimen was determined by the following formula:

(1) $$\varepsilon_{pl}(t_{i})=\frac{d_{s}-d_{s}(t_{i})}{d_{s}}\cdot 100$$

where $d_{s}$ is the length of the measuring base of the specimen and $d_{s}(t_{i})$ is the length of the measuring base of the specimen at the time of “rest” $t_{i}$ after the complete removal of the compressive stress.

Short-term compression and residual deformation tests were carried out at ambient temperature (23 ± 2) °C and (50 ± 5)% relative humidity. The test specimens of EPS were conditioned at ambient temperature (23 ± 2) °C and (50 ± 5)% relative humidity throughout the duration of the experiment.

The density of EPS is the main parameter determining its properties under static loading; thus, the results of the investigation of plastic deformation are presented according to this basic parameter at fixed active deformations $\varepsilon_{i}$ in the range from 15 to 65%. The macro and microstructure investigations were performed using the electron scanning microscope JEOLJSM-7600F.

For the processing of the experimental data received, the mathematical-statistical methods along with the program package (Statistica) were used [33].

The studied values of residual (plastic) strains are scattered randomly around a mean value $\bar{Y}$. This scattering reveals the stochastic nature of the relationship between the values determined by these test methods. Therefore, regression analysis is expedient to find the relationship Y=f(X) and to determine the values Y based on measured or fixed values of parameters X, such as density ρ or fixed level of deformation $\varepsilon_{i}$ during loading. The experimental data were processed with a confidence probability of p=0.90 with one-sided criteria. Preliminary control of the experimental data “on the anomaly” was established under the assumption of a one-dimensional measurement system. Relationships with sufficient accuracy over a wide range of EPS densities were used to determine the predicted values of residual (plastic) strain $\varepsilon_{pl}^{\left(1\right)}$ after full unloading (Table 1). They were approximated by an empirical equation:

(2) $$\bar{\varepsilon_{pl}^{\left(1\right)}}=b_{0}\rho^{b_{1}}e^{b_{2}\cdot \rho }$$

where $\bar{\varepsilon_{pl}^{\left(1\right)}}$ is the average value of the residual (plastic) strain of the specimen immediately after removing the load, which corresponds to the active fixed deformation, %; ρ is the density of the specimen, kg/m^3^; and $b_{0},b_{1}$, and $b_{2}$ are the constant parameters, which are calculated from the experimental data using the least squares method [34].

The degree of relationship between two variables in the regression scheme with a nonlinear dependence is characterized by the correlation ratio $\eta_{y\cdot x}$. The portion of the variation of the test characteristic that depends on the variation of the concrete controlled input factor is shown by the coefficient of determination. In the presence of a nonlinear relationship, the coefficient of determination is the square of the correlation ratio $\eta_{y\cdot x}^{2}$ [35]. Because a measure of the scattering of test results around the regression line was accepted, the standard deviation $S_{r}$, which is the absolute value of the mean test data deviation from the empirical regression line, was invariable for all sections of this line:

(3) $$S_{r}=\sqrt{\frac{\sum_{i=1}^{i=n}{\left(Y_{x_{i}}-{\bar{Y}}_{x_{i}}\right)}^{2}}{n-m}}$$

where $Y_{x_{i}}$, ${\bar{Y}}_{x_{i}}$ is experimental and calculated by the empirical equation for the i-th value of the resultant, n is the number of test results, and m is the number of estimated parameters in the empirical equation.

In addition to the predicted resultant as a single numerical value (a point value of prediction ${\bar{Y}}_{x_{i}}$), the possible value of error δ, which allows the method to switch to interval prediction, was calculated:

(4) $$Y_{x_{i}}^{pred.}={\bar{Y}}_{x_{i}}\pm \delta$$

where ${\bar{Y}}_{x_{i}}$ is the point value of the prediction based on, for example, the empirical Equation (2). According to [36]:

(5) $$\delta =t_{\alpha ;f}\cdot S_{r}$$

where $t_{\alpha ;f}$ is the Student’s criterion, which is chosen as the confidence probability of the prediction p=0.90 (with a one-sided criterion) and depends on the number of degrees of freedom f=n−m [37].

The unilateral maximum and minimum limits of the confidence interval for the predicted assessment of resultant indication are $\left({\bar{Y}}_{x}+\delta \right)$ and $\left({\bar{Y}}_{x}-\delta \right)$, respectively.

### 2.3. EPS Characterization

Commercially available EPS slabs are characterized by a broadly varying density, such as from 12 kg/m^3^ to 40 kg/m^3^, based on their EPS type.

**Figure 1:**
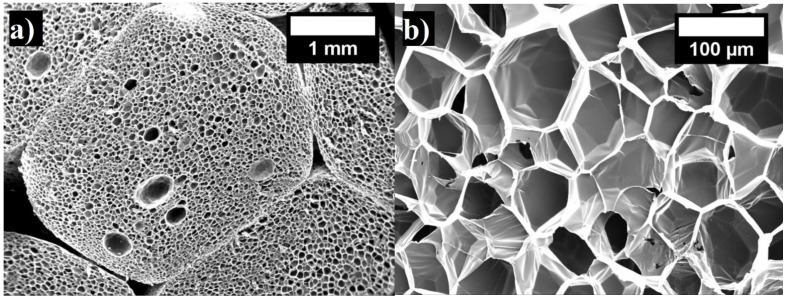
The presented structure of EPS at 17 kg/m^3^ density: (**a**) microstructure of pores (magnification ×80); (**b**) microstructure of cells (magnification ×800).

Therefore, the whole range was selected and all specimens were divided into two groups: 12–22 kg/m^3^ and 22–40 kg/m^3^. Two density ranges were selected in order to evaluate their impact on residual deformations. The structure of the EPS specimen is presented in Figure 1, and the main properties for further testing are shown in Table 1.

## 3. Results and Discussion

**Figure 2:**
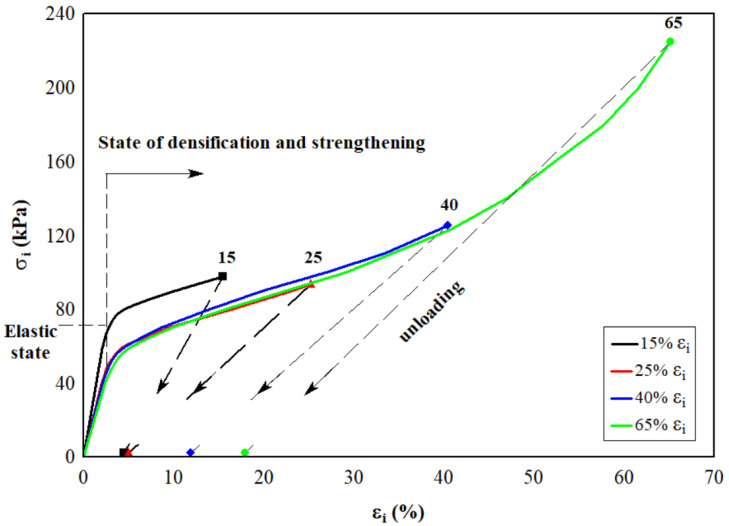
The elastic–plastic behavior of EPS with a density of 14–18 kg/m^3^ at short-term compression. Points along the *x*-axis are experimental values of residual strain after full unloading of the EPS specimens.

Under compression, the stress continuously grows and exceeds the elastic limit [6,38], and the relationship between stress $\sigma_{i}$ and strain $\varepsilon_{i}$ is characterized by the curves shown in Figure 2. Their relationship depends on changes that emerge in the macrostructure [38,39] with loading. Three regions exist in the stress–strain diagrams: a steep, quasi-linear initial section, which corresponds to an increase in the load in the range of small strains that do not exceed 3% (the stress varies from zero to 80 kPa); a section with a much greater range of deformation changes with a relatively small (25–30%) increase in stress, which corresponds to hardening of the EPS; and a section with further loading, which corresponds to compression of the compacted EPS. These curves can be described by $\sigma_{i}=\phi (\varepsilon_{i})$. Once the EPS specimen entered into the elastic–plastic state ($\varepsilon_{i}$, $\sigma_{i}$) and fully discharged, its deformation was partly saved; for example, diagrams of discharging $\sigma_{i}$–$\varepsilon_{i}$ are different from the diagrams shown in Figure 2. This plastic deformation is in contrast to an elastic deformation [40] and is an irreversible process. Some of the external forces change the structure of the specimen during loading, such as the conversion of the shift in slip planes into thermal energy. Furthermore, similar observations in the stress–strain curves have been made by Ling et al. [41] at different EPS densities.

**Figure 3:**
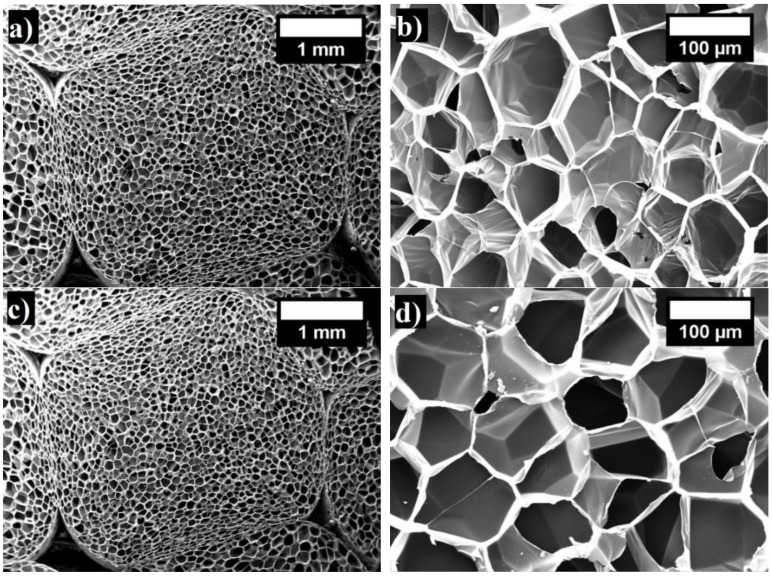

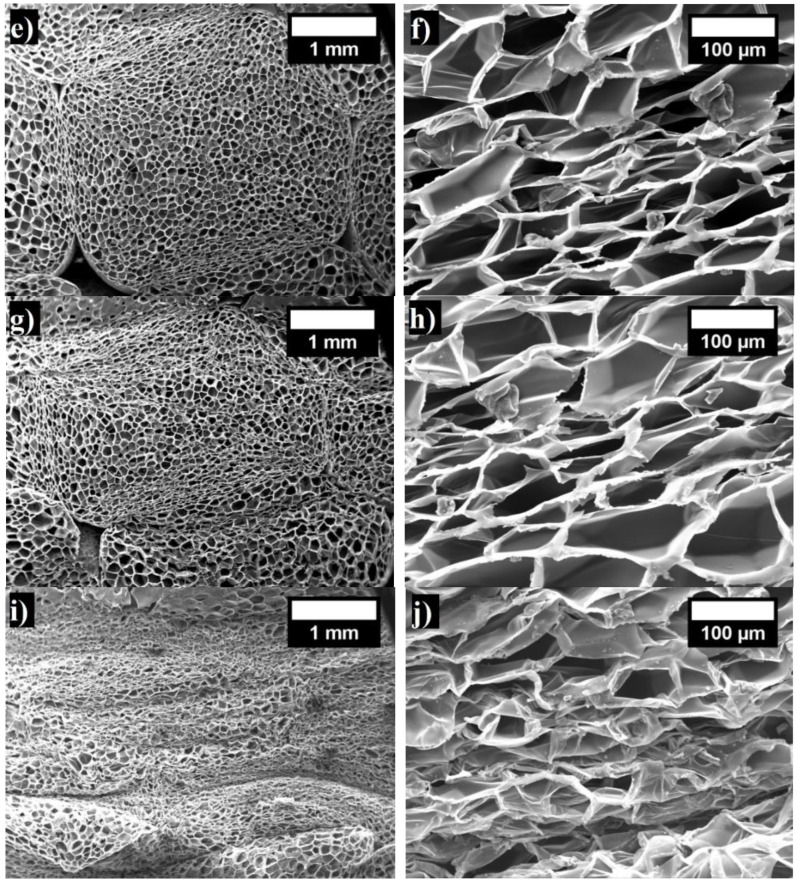
The changes of the 17 kg/m^3^ density EPS specimen structure in the places of pore and cell connections under different levels of compression, %: (**a**) 0 (magnification ×80); (**b**) 0 (magnification ×800); (**c**) 3 (magnification ×80); (**d**) 3 (magnification ×800); (**e**) 10 (magnification ×80); (**f**) 10 (magnification ×800); (**g**) 30 (magnification ×80); (**h**) 30 (magnification ×800); (**i**) 60 (magnification ×80); (**j**) 60 (magnification ×800).

The structure of the EPS, depending on the density of the material, is characterized by closed pores of a certain size and the arrangement of the pores in the product. The EPS slabs are made up of a hard polymeric matrix and a gaseous phase. The gaseous phase of EPS materials can take up to 98% of their volume. Whereas cellular materials are classified according to their cellular structure and cell connectivity, unlike polyurethane foam [42], EPS is always characterized by a closed-cell structure. The changes in the EPS structure that take place under different levels of compression are presented in Figure 3. In Figure 3a,b, the structure of the non-deformed specimen can be seen, and a few interconnected pores and row-spacings between them are visible [43]. When the deformation reaches 3%, the spaces between the pores diminish only slightly (Figure 3c,d). The EPS pores move towards free space, and the structure of the pores undergoes almost no changes. Figure 3e,f presents the EPS specimen that is compressed up to 10%.

The EPS pores are deformed at the places where they connect with each other (Figure 3e,f). For the EPS specimen at a compression of 30% relative deformation, a decrease of the spaces between pores occurs and progressive deformations of the connected pore walls and cells appear (Figure 3g,h). When reaching a compression of 60% deformation, substantial changes occur, and the collapsing process of the EPS pores and cells appears (Figure 3i,j).

**Figure 4:**
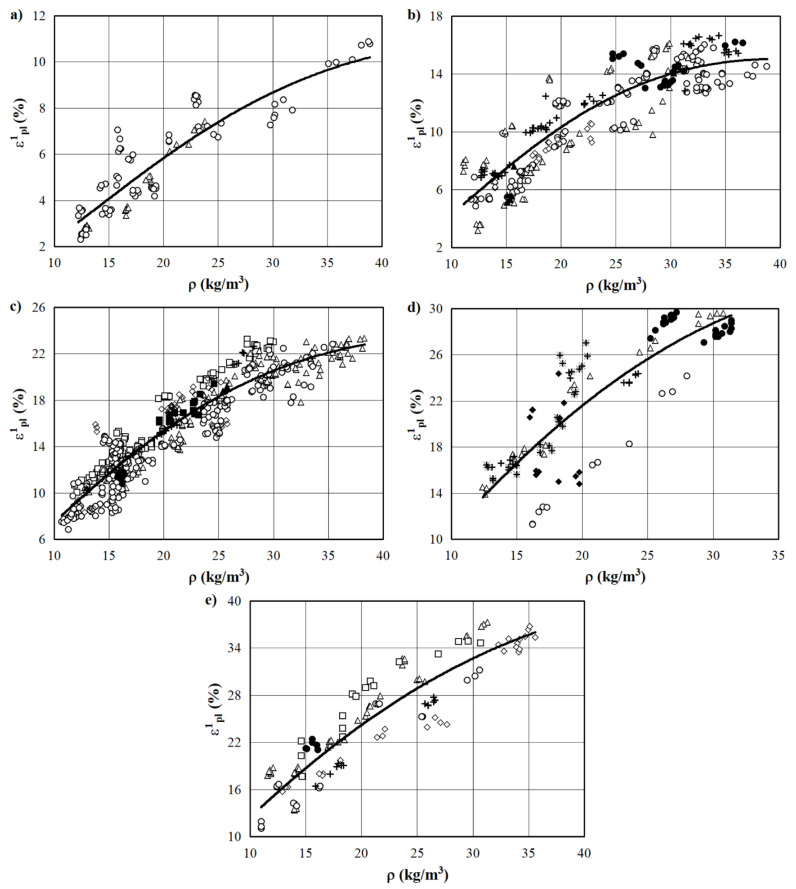
Residual (plastic) deformation of EPS $\varepsilon_{pl}^{\left(1\right)}$ immediately after the complete removal of the load. $\varepsilon_{i}$, %: (**a**) 20; (**b**) 30; (**c**) 40; (**d**) 50; (**e**) 60. (—) approximation of Equation (2); points ○, ∆, ⁪, ◊, ■, +, ●, ▲, ♦, and ⁕ are experimental values for EPS of different manufacturers.

The presence of an irreversible deformation is a physical symptom of an elastic–plastic state. The experimental values of the residual (plastic) deformation $\varepsilon_{pl}^{\left(1\right)}$ immediately after the complete unloading of specimens are presented by points along the *x*-axis in Figure 2. Residual (plastic) deformation $\varepsilon_{pl}^{\left(1\right)}$ of the EPS immediately after the complete removal of the applied load with an active deformation $\varepsilon_{i}$ equal to 20, 30, 40, 50, and 60%, depending on the density of the specimen, is presented in Figure 4.

The results of the statistical processing of the experimental data of the deformation $\varepsilon_{pl}^{\left(1\right)}$ are presented in Table 2. It should be noted that the coefficients of determination of the regression equations in the form of (2) range from 0.744 to 0.986 and, at a confidence level of 90%, are much higher than the threshold (lower limit) values of $\eta_{\varepsilon_{pl}^{\left(1\right)}\cdot \rho }^{2}$ for the corresponding n values [36]. Thus, the presented regression equations can be used to predict the values of the residual plastic deformation $\varepsilon_{pl}^{\left(1\right)}$ of commercially available EPS with different densities subjected to short-term compression.

Therefore, the value of the coefficient of determination after the active deformation to $\varepsilon_{i}=15\%$ indicates that 84% of the variation in the values of $\varepsilon_{pl}^{\left(1\right)}$ is caused by changes in density and that only 16% is caused by other factors [34] (see regression equation for $\varepsilon_{i}=15\%$ in the first line of Table 2).

**Figure 5:**
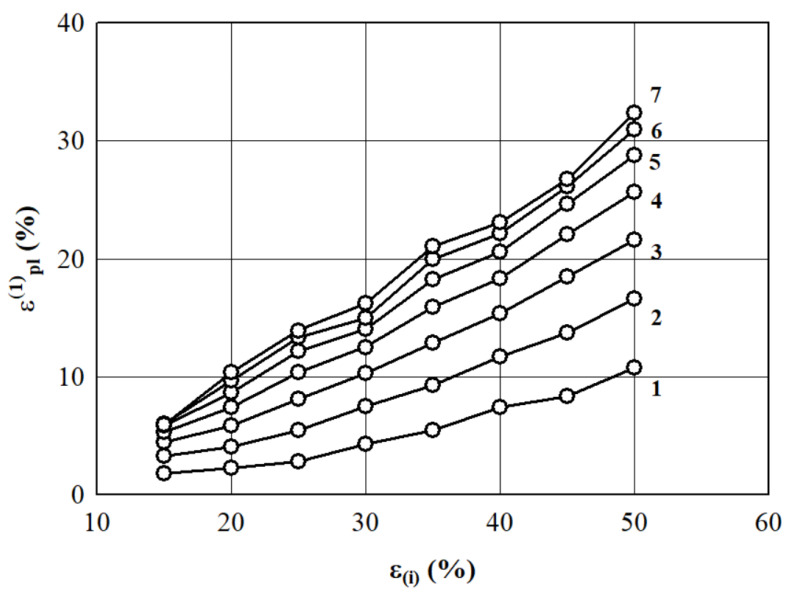
The dependence of the prognostic point values of residual (plastic) strain $\varepsilon_{pl}^{\left(1\right)}$ (calculated according to Equation (2)) on the value of active fixed deformation $\varepsilon_{i}$ when EPS density is 1–10 kg/m^3^; 2–15 kg/m^3^; 3–20 kg/m^3^; 4–25 kg/m^3^; 5–30 kg/m^3^; 6–35 kg/m^3^, and 7–40 kg/m^3^.

Point values of the deformation $\varepsilon_{pl}^{\left(1\right)}$ of EPS specimens with nominal densities of 10, 15, 20, 30, 35, and 40 kg/m^3^, which were calculated using the regression equations presented in Table 2, are shown in Figure 5. Ehinger et al. [44] wrote that the increase in strain or strain rate could affect the behavior of foam in different ways, including the strain or strain rate sensitivity. Therefore, according to the presented dependencies, it should be noted that the relationship is almost linear up to a fixed active deformation $\varepsilon_{i}$ of 50%, which corresponds to a proportional increase in the residual (plastic) deformations $\varepsilon_{pl}^{\left(1\right)}$. This observation of the current study is in great agreement with the findings of Chernous and Shil’ko [45]. For the fixed values of active deformations $\varepsilon_{i}>50\%$, this pattern of change $\varepsilon_{pl}^{\left(1\right)}$ is violated. It is rightly assumed that this change results from irreversible structural changes of the EPS, such as the final destruction of cells (with the exception of specimens with a density of 40 kg/m^3^, for which the maximum value $\varepsilon_{i}\geq 60\%$).

**Figure 6:**
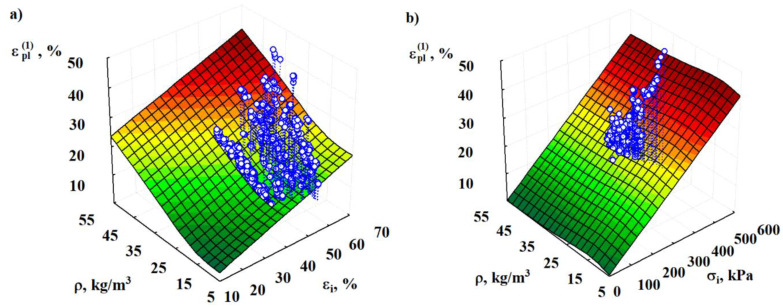
Graphical interpretation of the regression Equations (6) and (7) characterizing the dependence of the residual (plastic) deformation $\varepsilon_{pl}^{\left(1\right)}$ of EPS of various densities ρ on the values of the active deformation $\varepsilon_{i}$ (**a**) and compressive stress $\sigma_{i}$ created during the active deformation (**b**).

Xi et al. [46] proposed a model with temperature based on the experimental results and generated a static compression constitutive model. However, this model is dedicated to aluminum foams and takes into consideration the temperature effect. To assess the predicted values of residual deformation $\varepsilon_{pl}^{\left(1\right)}$, density variation ρ (kg/m^3^), active strain $\varepsilon_{i}$, and compressive stress $\sigma_{i}$ are evaluated and the dependencies are shown in Figure 6.

According to the coefficient of determination, the suggested regression is the most acceptable for the model in Figure 6a:

(6) $$\varepsilon_{pl}^{\left(1\right)}=68.24\exp\left(-\frac{65.9}{\rho }\right)+0.303\varepsilon_{i}$$

with a standard deviation $S_{r}=3.28\%\;(n=1710)$. The value of the latter suggests that the variation of $\eta_{\varepsilon_{pl}^{\left(1\right)}\cdot \rho \varepsilon_{i}}^{2}=0.779$ by 78% is caused by the change in density and the value of the fixed deformation $\varepsilon_{pl}^{\left(1\right)}$ during the active deformation.

The dependence of the deformation $\varepsilon_{pl}^{\left(1\right)}$ (%) on the density of specimens ρ (kg/m^3^) and compressive stress, $\sigma_{i}$ (kPa), of active deformation may be approximated by the following regression equation (Figure 6b):

(7) $$\varepsilon_{pl}^{\left(1\right)}=0.0052\rho \exp\left(-0.22\rho \right)+0.069\sigma_{i}$$

with a standard deviation $S_{r}=3.71\%\;(n=1710)$ and a total coefficient of determination $\eta_{\varepsilon_{pl}^{\left(1\right)}\cdot \rho \sigma_{i}}^{2}=0.717$. The variation of values $\varepsilon_{pl}^{\left(1\right)}$ by 72% is caused by the change in density ρ and stress $\sigma_{i}$. The experimental values $\varepsilon_{pl}^{\left(1\right)}$ for the specimens of each series are given in Table 1. It was determined that the “rest” of the EPS specimens for at least 205 days at ambient temperature (23 ± 2) °C results in a steady state, which is characterized by the presence of irreversible plastic deformation. Based on the decrease (reversibility) of the plastic deformation, $\alpha =\frac{\varepsilon_{pl}^{\left(1\right)}}{\varepsilon_{pl}^{\left(\tau \right)}}$, it should be noted that the plastic deformation $\varepsilon_{pl}^{\left(\tau \right)}$ (at τ≥205 days) is actually an irreversible plastic deformation.

Then, according to Table 1, the average ratio $\bar{\alpha }=1.06$, $\left(n=873;S_{\alpha }=0.049\right)$ for a density of EPS between 12 and 22 kg/m^3^, and $\bar{\alpha }=1.04$, $\left(n=629;S_{\alpha }=0.029\right)$ for a density between 23 and 40 kg/m^3^. This result demonstrates a partial reduction of the deformation $\varepsilon_{pl}^{\left(1\right)}$, for example, a partial restoration of the height of the specimens takes place due to the elastic aftereffect [37].

**Figure 7:**
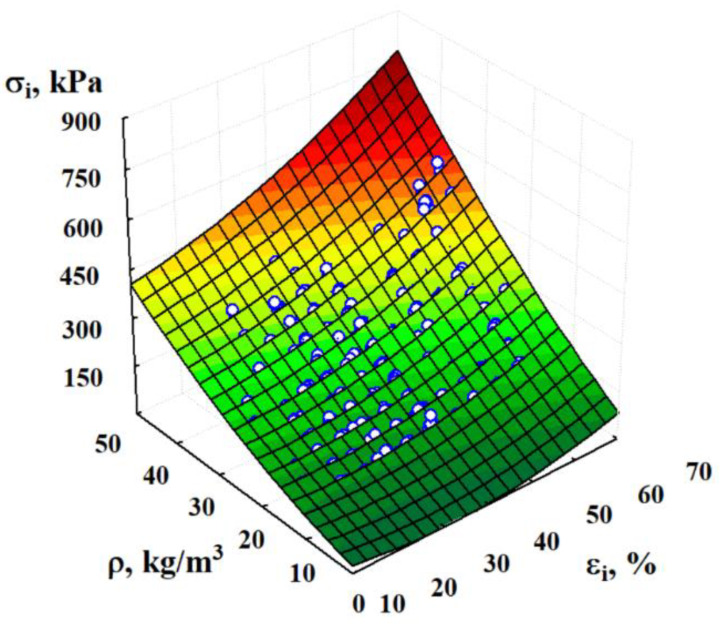
Graphical interpretation of the regression multinomial (8) characterizing the dependence of the stresses $\sigma_{i}$ on active elastic–plastic deformations $\varepsilon_{i}$.

The process of active deformation was analyzed, and the results were presented to demonstrate that the elastic limit exceeds the current values of stress $\sigma_{i}$ and strain $\varepsilon_{i}$ at the point of unloading (values of $\sigma_{cr}$ and $\varepsilon_{cr}$ [25,37]). The curve of the stress–strain relation in the whole range of active elastic–plastic deformation can be represented by a two-part relationship. The first section presents the elastic state $\sigma_{i}=E\cdot \varepsilon_{i}$, $\;0\leq \varepsilon_{i}\leq \varepsilon_{cr}$. It is quasi-linear and corresponds to elastic deformation, characterized by the value of the elastic modulus E [37]. The regressive description of this section in the range of strains $\varepsilon_{i}$ 0–35% was done in Ref. [38]. This section addresses the hardening curve with a variable modulus of elasticity. This curve is based on experiments with a simple monotonic increase in loading (in the range of strains from 15 to 65%), which may be approximated by a polynomial (Figure 7).

(8) $$\sigma_{i}=54.83-3.581\cdot \varepsilon_{i}+2.749\cdot \rho +0.0567\cdot \varepsilon_{i}^{2}+0.0771\cdot \rho^{2}+0.1051\cdot \varepsilon_{i}\cdot \rho$$

with a standard deviation $S_{r}=13.8$ kPa (n=1710) and the total coefficient of determination $\eta_{\sigma_{i}\cdot \varepsilon_{i}\rho }^{2}=0.974$ (where $\sigma_{i}$ (kPa); $\varepsilon_{i}$ (%); ρ (kg/m^3^)). The curve (8) passes through the point $\varepsilon_{i}=10\%$, $\sigma_{i}=\sigma_{10\%}$, which may slightly deviate from the range of studies ($\varepsilon_{i}$ from 15 to 65%). The error associated with the deviation (outside the observation range, the form and sometimes the direction of the relation may change) is small and is covered by the confidence interval (Equation (4)). Alaneme et al. [47] showed that aluminum foam is similar to EPS with regard to the manner of deformation and mechanical response. The authors have determined a very similar polynomial regression with high correlation rates, which allowed for the accurate description of stress results based on density and strain variations.

## 4. Conclusions

It is determined that EPS passes three stages during compression and the final destruction of cells occurs at 50% of active deformation. Moreover, it is shown that the residual deformation is dependent on the apparent density. The relation is described by the regression equation, which allows the prediction of residual deformation at a density range from 12 to 40 kg/m^3^. The obtained results also show that the residual deformation at the specified density range increases from 2% to 40% when the active deformation increases from 15% to 60%. Switching parameters in multicriteria regression analysis show that the apparent density, active deformation, and compressive stress are the most important factors in the proper prediction of residual deformation. Compared to previous modeling attempts, the proposed regression models allow for the accurate prediction of the residual deformation at the apparent density range of 12–40 kg/m^3^, active deformation 15–65%, and compressive stress 80–230 kPa. The implementation of the proposed models makes it possible to accurately determine the appropriate characteristics of EPS during compression, which makes it possible to increase the accuracy and effectiveness of the simulation. The research carried out and the results obtained allow for a better selection of the EPS type and its properties for the corresponding application.

## Figures and Tables

**Table 1:** Initial characteristics of EPS specimens for active and passive deformation after the short-term compression test and experimental values of residual (plastic) strain.

Test Series No.	Initial Characteristics of EPS Specimens before Testing			Test Conditions	
	Mean Apparent Density *ρ*, kg/m^3^	Mean Compressive Stress *σ_i_*, kPa	Mean Modulus of Elasticity *E*, MPa	Strain under Compressive Loading *ε_i_*, %	Time after Unloading *τ*, Days
1	16.3 ± 0.9 ^1^	86.2 ± 7.3 ^1^	3.26 ± 0.4 ^1^	15	647
	26.8 ± 0.6	171 ± 4.9	7.35 ± 0.2		
2	16.1 ± 0.6	84.6 ± 4.9	3.18 ± 0.2	20	288
	28.0 ± 2.3	181 ± 19	7.82 ± 0.9		
3	16.5 ± 1.1	87.8 ± 8.9	3.34 ± 0.4	25	645
	28.7 ± 1.6	187 ± 13	8.09 ± 0.6		
4	16.2 ± 0.4	85.4 ± 3.2	3.22 ± 0.2	30	435
	29.7 ± 0.7	195 ± 5.7	8.48 ± 0.3		
5	17.4 ± 0.4	95.1 ± 3.2	3.69 ± 0.2	35	287
	29.4 ± 0.9	192 ± 7.3	8.37 ± 0.4		
6	16.5 ± 0.3	87.8 ± 2.4	3.34 ± 0.1	40	422
	28.4 ± 0.6	184 ± 4.9	7.98 ± 0.2		
7	15.2 ± 1.0	77.3 ± 8.1	2.83 ± 0.4	45	707
	27.9 ± 2.1	180 ± 17	7.78 ± 0.8		
8	16.8 ± 0.7	90.3 ± 5.7	3.45 ± 0.3	50	588
	27.6 ± 0.8	178 ± 6.5	7.66 ± 0.3		
9	15.4 ± 0.9	78.9 ± 7.3	2.91 ± 0.4	55	205
	30.2 ± 1.3	199 ± 11	8.68 ± 0.5		
10	16.3 ± 0.7	86.2 ± 5.7	3.26 ± 0.3	60	863
	28.6 ± 1.1	186 ± 8.9	8.05 ± 0.4		
11	20.4 ± 0.9	119 ± 7.3	4.86 ± 0.4	65	244
	30.9 ± 3.6	204 ± 29	8.95 ± 1.4		

^1^ Confidence estimate with reliability, *p* = 0.90.

**Table 2:** The results obtained in the regressive analysis of the experimental values of the plastic strain $\varepsilon_{pl}^{\left(1\right)}$ immediately after the complete unloading of specimens subject to the elastic–plastic state (*ε_i_*, *σ_i_*).

Test Series No.	Strain under Compressive Loading, %	Number of Tested Specimens	Parameters of Equation (2)			Coeff. of Determination $\eta_{\varepsilon_{pl}^{\left(1\right)}\cdot \rho }^{2}$	Standard Deviation $S_{r},\%$	Possible Error δ, %
			$b_{0}$	$b_{1}$	$b_{2}$			
1	15	48	0.0233	2.1585	−0.0605	0.835	0.490	0.637
2	20	96	0.0422	1.8928	−0.0370	0.821	0.932	1.204
3	25	109	0.0241	2.2900	−0.0522	0.866	1.145	1.477
4	30	316	0.0721	1.9927	−0.0502	0.811	1.537	1.974
5	35	375	0.1343	1.7680	−0.0367	0.910	1.251	1.606
6	40	570	0.3232	1.5000	−0.0316	0.871	1.523	1.953
7	45	31	0.2240	1.7547	−0.0423	0.970	0.836	1.098
8	50	110	0.5428	1.4246	−0.0292	0.744	2.851	3.676
9	55	96	0.5921	1.2666	−0.0146	0.914	2.162	2.791
10	60	125	0.8236	1.2736	−0.0217	0.833	2.905	3.745
11	65	20	0.0617	2.4401	−0.0605	0.986	1.286	1.714

## Data Availability

Not applicable.
